# Peltier Supercooling in Transient Thermoelectrics: Spatial Temperature Profile and Characteristic Cooling Length

**DOI:** 10.3390/e21030226

**Published:** 2019-02-27

**Authors:** Pablo Eduardo Ruiz-Ortega, Miguel Angel Olivares-Robles

**Affiliations:** 1 Instituto Politecnico Nacional, Depto. Ingenieria Bioquimica, ENCB, Ciudad de Mexico 07738, Mexico; 2 Instituto Politecnico Nacional, SEPI ESIME Culhuacan, Ciudad de Mexico 04430, Mexico

**Keywords:** supercooling, thermoelectric, Peltier, pulse current, transitory

## Abstract

Thermoelectric coolers (TECs) can reach temperatures below that obtained with a steady-state current by applying an electrical current pulse which enables a transitory state in a Peltier device. This effect is known as supercooling. In this paper, we study characteristics parameters, such as the minimum cooling temperature and spatial temperature profile, in a TEC operated under current pulses and a cooling load $\left(Q_{c}\right)$. Numerical analysis for a one-dimensional thermoelectric model of the cooling system is developed, and a novel MATLAB programming code is proposed for the transient state based on finite element analysis. We also investigate the influence of the thermoelement’s length upon the cooling mechanism. A new parameter called the “characteristic cooling length” is proposed to describe the length in which the minimum cooling temperature occurs along the elements of a TEM. Results show the transient temperature profiles along the elements of the semiconductor P-type element, and a “characteristic cooling length” is characterized. We also propose a general principle, and the lowest cooling temperature values are obtained for a semiconductor’s small length and variable pulse cooling load under current pulse operation. The present study will serve as guidance for the geometric design of TECs under current pulse operations.

## 1. Introduction

Thermoelectric cooling devices use electricity to generate a temperature gradient based on the Peltier effect. Operation of thermoelectric coolers (TECs) consists of establishing continuous temperature and voltage gradients along a thermoelectric element while maintaining the local thermodynamic equilibrium [1]. Thermoelectric effects, such as the Seebeck effect, Peltier effect, and Thomson effect, result from the interference of electrical currents and heat flow in the semiconductor’s material, and its interaction allows the usage of thermoelectric effects for cooling phenomena [2]. It is well-known that there exists an optimum electrical current $\left(I_{opt}\right)$ for a thermoelectric module (TEM) to generate a maximum temperature difference under a steady current operation [3]. TECs can reach temperatures below that obtained with a steady-state current by applying an electrical current pulse which enables a transitory state in a Peltier thermocouple. This is known as supercooling [4]. When a pulse current is applied to a device operating at a steady-state current, the cold side temperature drops immediately to lower values compared to the steady-state current operation, because of the Peltier effect. Conversely, supercooling is followed by an increase of the cold side temperature to a peak overshoot value due to accumulation of Joule heat in the thermoelement, as well as Fourier heat conduction through the thermoelement from the hot side to the cold side [5]. This phenomenon occurs because Peltier cooling, at the cold end of the thermoelectric material, is an instantaneous and interfacial effect that only takes place at the cold junction of each couple; while Joule heating, which occurs uniformly across the bar, requires more time to reach the interface due to the finite thermal diffusion rate, which leads to a temporary temperature drop on the interface. During the current pulse, the cooling at the cold end occurs before the Joule heat reaches this end, and a temporary temperature drop can be observed [6]. The coefficient of performance (COP) of thermoelectric devices is dependent on the semiconductor property materials used, and the number and geometry of the thermoelements. Several new theoretical and practical methods for the improvement of cooling devices have been studied and, at last, significant advances are being made [7]. Snyder et al. numerically and experimentally studied the pulse thermoelectric cooling and showed that this technique can provide low cold side temperature which is comparable to the cold side temperature of a two-stage thermoelectric cooler [8]. Yang et al. derived the expressions for the time to reach the minimum temperature and the holding time with the pulse operation of a thermoelectric cooler [9], and Meng et al. [10] compared the dynamic temperature variations at the cold and hot ends using a three-dimensional transient TEC model. Manikandan et al. studied a modified pulse operation method using the hot side heat transfer coefficient—also pulsed—and showed an improvement in the efficiency, being 23.3% higher than a normal pulse operation [11]. The three-dimensional theoretical model has also been studied, where Chen et al. developed a simulation model to predict transient thermal behavior of the thermoelectric coolers [12]. Shen et al. [13] and others [14,15,16] have theoretically and experimentally studied and demonstrated the pulse operation of the thermoelectric coolers. Several studies about the development of thermoelectric devices have been made, and one of them includes a study by Lineykin [17] where a SPICE (Simulation Program with Integrated Circuits Emphasis) compatible equivalent circuit of a thermoelectric module was developed, which can be used to design feedback networks for temperature control applications. Possible applications of these devices have been investigated by Zhang et al. [18] where the performance of a TEC was studied for electronic packages such as processors using optimized currents and cooling configurations. Works at microscale by Koh et al. [19] analyzed thermoelectric (TE) microcoolers for hotspot cooling aiming for the development of TE devices. The transient supercooling effect can find many applications where extra cooling required for a short time, such as infrared detectors during the winking mode, and condensation hygrometers require a significant decrease in temperature to reach the dew point, as well as supercooling the nozzles or tubes in a conventional refrigerator.

Transient cooling was investigated by modeling the heat and electricity conductors, and we considered thermoelectric effects during pulse operation in order to predict the most important parameters where extra cooling was needed for a short time. The Peltier effect and Joule heating play an important role in the transient supercooling effect, and must be investigated to find new applications or improve some of them, such as infrared detectors or tubes in a conventional refrigerator [20]. This is an advantage of using transient state models for better characterization of supercooling, compared to the steady-state model where lower temperatures cannot be reached. Therefore, in this work, we propose a parameter to establish optimal conditions for a better super-cooling method. In this work, we will discuss the influences of operating condition parameters on temperature profiles. However, it should be noted that a characteristic cooling length appears when a variable cooling load is considered together with the current pulse. We propose a general principle to make a judgment on whether a temperature profile will occur during or after a current pulse.

## 2. Thermodynamic Modelling for Pulse Operation of Thermoelectric Cooler

**Figure 1:**
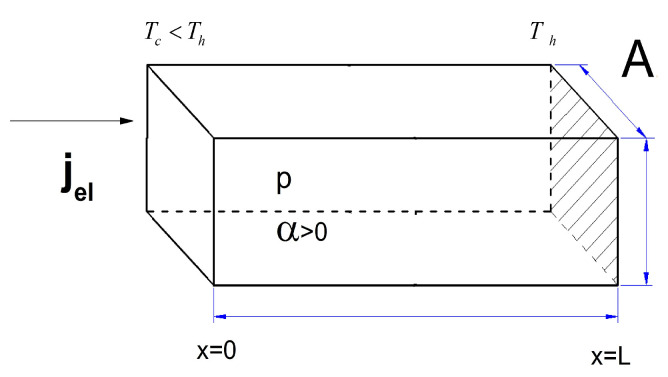
1D model of a p-type pellet of a Peltier element.

A thermoelectric cooler is generally composed of several semiconductor element pairs—p-type and n-type—connected electrically in series and thermally in parallel. In our work, we develop a numerical model based on the finite element method to study the transient characteristic of the temperature profile of a P-type semiconductor element. As it is well-known, the thermal features and temperature distributions in TE elements are almost the same and periodic—hence, only one element is considered in the modeling. Numerical solutions for the one-dimensional temperature profiles in the P-type semiconductor element and transient variations in the cold side temperature are carried out. The numerical analysis of the cooling pulse is solved for the one-dimensional model shown in Figure 1.

The thermoelectric material properties and the dimensions of the thermoelectric element are given in Table 1 [3].

## 3. Transient State Equations

The governing equations for one-dimensional unsteady state heat transfer by applying an energy balance analysis for the P-type element leads to [12]:

(1) $$\frac{\partial^{2}T}{\partial x^{2}}-\frac{I\tau }{A\kappa }\frac{\partial T}{\partial x}+\frac{I^{2}\varepsilon }{A^{2}\kappa }=\frac{\rho C_{p}}{\kappa }\frac{\partial T}{\partial t}$$

where *T* is the temperature, κ is the thermal conductivity, *t* is the time, τ is the Thomson effect, ε is the electrical resistivity, ρ is the density, *A* is the cross-sectional area of the semiconductor element, *I* is the electrical current, and $C_{p}$ is the specific heat. The boundary conditions on the surfaces of the P-type element is as follows [16]:

(2) $$\kappa \frac{\partial T}{\partial x}{|}_{x=0}=\frac{Q_{c}}{A}=q_{c}\;\kappa \frac{\partial T}{\partial x}{|}_{x=L}=T_{h}$$

The hot end of a TEC is generally attached to a heat sink, and hence in this work, a boundary condition was applied to the hot end with a temperature of $T_{h}$ =300 K. The cold end of the TEC was attached to a surface with a uniform heat flux. Thus, a constant heat flux boundary condition was applied to the cold end, which is convenient for the present model to study the effect of pulse current and pulse cooling load.

### 3.1. Normalized Pulses Magnitude

In this work, the supercooling effect is described as a function of the independent variables—the pulse–current ratio and pulse cooling load ratio, which are defined as:

(3) $$P_{r}=\frac{I_{pulse}}{I_{steady}}$$

(4) $$Q_{r}=\frac{Q_{pulse}}{Q_{steady}}$$

### 3.2. Equation Solution by Finite Element Method

We used the Petrov-Galerkin method [21] to develop a numerical solution to the problem. The governing equations were discretized based on the finite-difference method. First, an approximation for the temperature profile was proposed $T≃T^{h}=T^{h}\left(x,t\right)$ and replaced in the main equation to obtain a residual error *r*, given by:

(5) $$r=\frac{\partial^{2}T^{h}}{\partial x^{2}}-\frac{I\tau }{A\kappa }\frac{\partial T^{h}}{\partial x}+\frac{I^{2}\varepsilon }{A^{2}\kappa }-\frac{\rho C_{p}}{\kappa }\frac{\partial T^{h}}{\partial t}$$

In the Petrov-Galerkin method, local subdomains were defined for each node, which was entirely within the global domain. Each of the subdomains is equal to zero—that is:

(6) $$\int_{\Omega }r\psi_{i}d\Omega =0$$

where $\psi_{i}$, the so-called expansion function corresponding to the i-th node, is a function that only depends on spatial coordinates, and Ω is the local subdomain which was analyzed. Substituting the Equation (5) into Equation (6), we have:

(7) $$\int_{\Omega }\frac{\partial^{2}T^{h}}{\partial x^{2}}\psi_{i}d\Omega -\int_{\Omega }\frac{I\tau }{A\kappa }\frac{\partial T^{h}}{\partial x}\psi_{i}d\Omega -\frac{\rho C_{p}}{\kappa }\int_{\Omega }\frac{\partial T^{h}}{\partial t}\psi_{i}d\Omega +\int_{\Omega }\frac{I^{2}\varepsilon }{A^{2}\kappa }\psi_{i}d\Omega =0$$

Next, the first term of the previous expression is integrated:

(8) $$\int_{\Omega }\frac{\partial^{2}T^{h}}{\partial x^{2}}\psi_{i}d\Omega =\int_{\Omega }\frac{\partial }{\partial x}\left(\frac{\partial T^{h}}{\partial x}\psi_{i}\right)d\Omega -\int_{\Omega }\frac{\partial T^{h}}{\partial x}\frac{\partial \psi_{i}}{\partial x}\psi_{i}d\Omega$$

applying Gauss’s theorem [22], we have:

(9) $$\int_{V}\frac{\partial }{\partial x_{i}}\lambda dV=\int_{S}\lambda n_{i}dS\;where\;\lambda =\frac{\partial T^{h}}{\partial x}\psi_{i}$$

we get:

(10) $$\int_{\Omega }\frac{\partial^{2}T^{h}}{\partial x^{2}}\psi_{i}d\Omega =\int_{\Gamma }\frac{\partial T^{h}}{\partial x}n_{1}\psi_{i}d\Gamma -\int_{\Omega }\frac{\partial T^{h}}{\partial x}\frac{\partial \psi_{i}}{\partial x}\psi_{i}d\Omega$$

where Γ is the border of the local domain Ω and $n_{1}$ is the component in the *x* direction of the unit normal vector *n*. Replacing the Equation (10) in Equation (7), we get the expression for the nodes, which are completely within the domain of the problem:

(11) $$\int_{\Omega }\left(\frac{\partial T^{h}}{\partial x}\frac{\partial \psi_{i}}{\partial x}-\frac{I\tau }{A\kappa }\frac{\partial T^{h}}{\partial x}\psi_{i}-\frac{\rho C_{p}}{\kappa }\frac{\partial T^{h}}{\partial t}\psi_{i}+\frac{I^{2}\varepsilon }{A^{2}\kappa }\psi_{i}d\Omega \right)d\Omega -\int_{\Gamma }\left(\frac{\partial T^{h}}{\partial x}n_{1}\psi_{i}\right)d\Gamma =0$$

The function $T^{h}\left(x,t\right)$ is expressed as a linear combination of the expansion functions, which are (a) ϕ(x) functions only of the spatial coordinates and (b) T(t) the values of the temperatures to be found, which will depend only on time—that is:

(12) $$T^{h}\left(x,t\right)=\sum_{j=1}^{n}T_{j}\left(t\right)\phi_{j}\left(x\right)$$

where *n* is the number of nodes neighboring a given node *i*, from j=1…n, with n=3 for the one-dimensional case. From Equation (12), we get the temporal derivatives as:

(13) $$\frac{\partial T^{h}}{\partial t}=\sum_{j=1}^{n}\frac{\partial T_{j}}{\partial t}\phi_{j}$$

and the spatial derivatives,

(14) $$\frac{\partial T^{h}}{\partial x}=\sum_{j=1}^{n}T_{j}\frac{\partial \phi_{j}}{\partial x}$$

Here, in this work $\phi_{i}=\omega_{i}=\omega \left(x\right)$ [22], where ω is the weight function and, by means of it, we can select the form of the subdomains of integration of the weak form and it is used for the construction of the expansion functions as it is used as a weight function. Replacing the Equations (12)–(14) into Equation (11), we get:

(15) $$\int_{\Omega }\left(\sum_{j=1}^{n}\frac{\rho C_{p}}{\kappa }\frac{\partial T_{j}}{\partial t}\phi_{i}+\frac{I^{2}\varepsilon }{A^{2}\kappa }-\sum_{j=1}^{n}\frac{I\tau }{A\kappa }T_{j}\frac{\partial \phi_{j}}{\partial x}\right)\omega_{i}d\Omega +\int_{\Omega }\sum_{j=1}^{n}\left(T_{j}\frac{\partial \phi_{j}}{\partial x}\frac{\partial \omega_{i}}{\partial x}\right)d\Omega -\int_{\Gamma }\sum_{j=1}^{n}\left(T_{j}\frac{\partial \phi_{j}}{\partial x}n_{1}\omega_{i}\right)d\Gamma =0$$

We can rewrite the expression (15) in the form of a system of linear equations:

(16) $$C\dot{T}+KT=f$$

where the matrix *C* contains the coefficients of the temporal derivatives:

(17) $$C_{i,j}=\int_{\Omega }\frac{\rho C_{p}}{\kappa }\phi_{i}\omega_{i}d\Gamma$$

the vector *f* contains the source term:

(18) $$f_{i}=\int_{\Omega }\frac{I^{2}\varepsilon }{A^{2}\kappa }\omega_{i}d\Gamma$$

the matrix *K* contains the coefficients of the spatial derivatives:

(19) $$K_{i,j}=\int_{\Omega }\left(\frac{I\tau }{A\kappa }\frac{\partial \phi_{j}}{\partial x}\omega_{i}+\frac{\partial \phi_{j}}{\partial x}\frac{\partial \omega_{i}}{\partial x}\right)d\Omega -\int_{\Gamma }\left(\frac{\partial \phi_{j}}{\partial x}n_{1}\omega_{i}\right)d\Gamma$$

and the vectors *T* and $\dot{T}$ are the unknown temperatures through time:

(20) $${\dot{T}}_{j}=\frac{\partial T_{j}\left(t\right)}{\partial x}\;and\;T_{j}=T_{j}\left(t\right)$$

A novel MATLAB code, based on the finite element method, was developed for the analysis and to obtain the temperature profiles of the P-type semiconductor. The developed algorithm was able to divide the system into local subdomains, which were analyzed individually to obtain the spatial information necessary to construct the matrices according to the boundary conditions provided for one-dimensional analysis. A MATLAB program was developed for a transient system capable of achieving steady-state conditions when an electric current is applied for a certain time. During the pulse-current operation, the program plots the temperature distribution curves along the thermoelement in time periods in order to analyze the supercooling. The MATLAB program provides control over the finite element method, thus making it easy to customize the program according to requirements such as leg geometry.

### 3.3. Cooling Power ($Q_{c}$)

According to the theory of non-equilibrium thermodynamics, the thermal flux can be calculated as:

(21) $$j_{q}\left(x\right)=-\kappa \frac{\partial T}{\partial x}+j_{el}^{0}\alpha T\left(x\right)$$

where $Q_{c}=j_{q}\left(0\right)A$ and the heat flow released ($Q_{h}=j_{q}\left(L\right)A$) can be calculated for the cold side (x=0) and the hot side (x=L), using the electrical current $I=j_{el}^{0}$ A.

## 4. Results and Discussion

**Figure 2:**
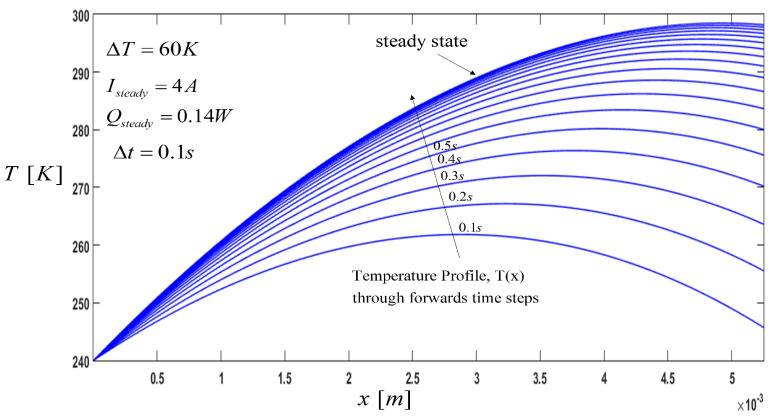
Temperature Profile, T(x), at a steady state along the thermoelement, where the direction of the arrow indicates the temperature profile through the time steps until reaching the steady state.

Temperature profiles during the pulse-current operation were studied, and the following cases were considered: (a) constant pulse, and (b) variable pulse of the cooling load. Our simulation model performed a study of the effects of the pulse current and pulse cooling load parameters. The relevant constant parameters in the computation are listed in Table 1. The numerical program for transient state can predict the steady-state operation of a TEC that works with constant electric current. Firstly, the current was imposed for a sufficient time to let the P-type element reach the corresponding steady state, as it is also reported in other works [11]. To obtain all the values needed to plot the curves, a temperature was first imposed along the thermoelement (240 K) with an electric current of $I_{steady}=4$ A. Then, the temperature distribution profiles were plotted as a function of the time, with time steps of Δt=0.1 s. For longer periods of time, temperature variation between curves showed an approximately parallel tendency, and after 1.8 s the system reached its steady state, as shown in Figure 2. The cooling load for steady-state operation was $Q_{c}=0.14$ W for a ΔT=60 K.

### 4.1. Temperature Profile: Constant Pulse of Cooling Load

**Figure 3:**
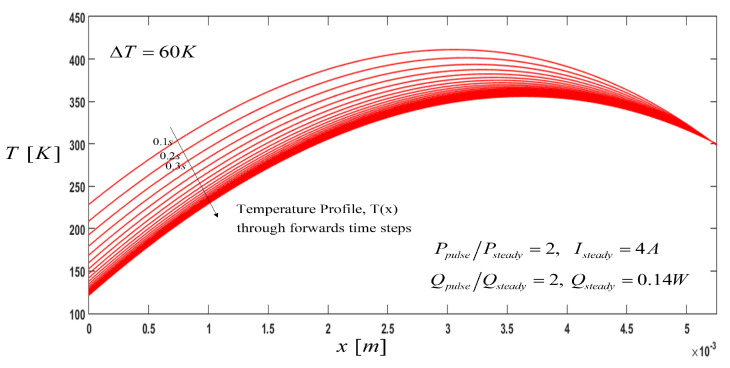
Temperature Profile, T(x): Effect of the pulse current and constant pulse-cooling load along the thermoelement, where the direction of the arrow indicates the temperature profile through the time-steps until reaching a steady state.

After the thermoelectric P-type semiconductor cooler reached its steady state, a pulse current ($I_{pulse}=8$ A) larger than the steady state current ($I_{steady}=4$ A) was applied to the system for a period of time. It is well-known from the literature [7] that the ratio $P=I_{pulse}/I_{steady}$ must be P>2. In addition to the pulse current, a constant cooling load pulse, $Q_{c,pulse}=0.34$ W, was also applied. When the current pulse and the cooling load pulse were applied, the temperature of the cold side dropped to a temperature of lower value than the temperature at the steady-state condition. Temperature profiles, along with the semiconductor, are shown in Figure 3 at different specific times. Each parabolic line curve represents the temperature profiles for every time-step of data, with Δt=0.1 s. It is noted that there exists a limit time to apply the pulse current to reach the minimum cooling temperature, which, in this case, is $T_{c_{min}}=124$ K after some time of Δt=4.2 s. The model was allowed to run to a quasi-steady state, and these results are in concordance with previous works where the only main difference is that in this work a pulse-cooling load was also considered. Therefore, Figure 3 shows the effect of various times between current pulses and its effect on the average cooled object’s temperature [8].

During the pulse, the temperature increases along the semiconductor element, reaching maximum temperature values at the start of the pulse current and decreasing with the time until it gets a minimum cooling temperature. We must note that the midpoint of the thermoelement reaches the maximum temperature due to increased volumetric Joule heating.

**Figure 4:**
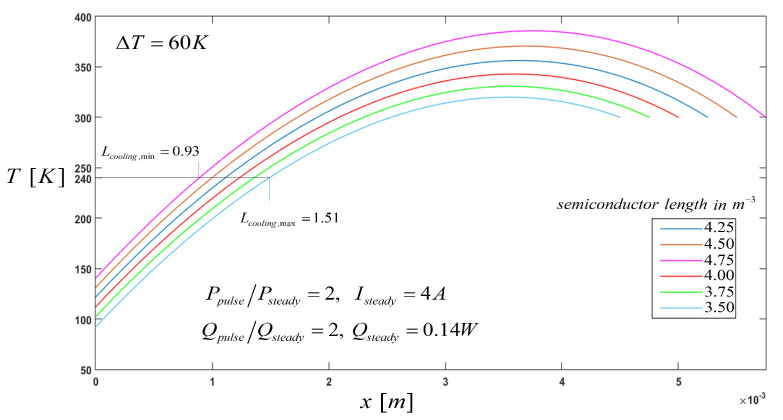
Temperature Profile, T(x), after the pulse current and constant pulse-cooling load along the thermoelement.

From the temperature profile reached in the element for the steady state, where $T_{c}=240$ K shown in Figure 2, samples of different lengths were analyzed during the pulse operation. From Figure 4, the minimum cooling temperature shows a dependence in function of the sample length, and this is what we call the characteristic cooling length, $L_{cooling}$. The cooling temperature decreases in the element during pulse operation, and it is shown that for the values of lowest length, $L_{cooling}$ increases. This characteristic length is the part of the semiconductor length which is cooled during pulse-current operation, and it can vary with the geometric parameters, cross-sectional area, and length. For the values of lowest length, $L_{cooling}$ increases, as shown in Figure 4. for a sample of $L=3.50\times 10^{-3}$ m we got a máximum value of $L_{cooling,max}=1.51$, compared to a sample of $L=4.75\times 10^{-3}$ m where we got a minimum value of $L_{cooling,min}=0.93$.

As it is well-known, according to the Seebeck effect, at the beginning it causes heat output from the cold end so that the temperature from the cold end is decreased. In addition, increasing the element length has two main effects on cooling temperature: (1) it increases the thermal resistance against heat conduction, and (2) increases electrical resistance as well as the Joule heating effects, meaning that the cooling temperature decreases.

This paper shows that the heat transport in a transient system depends strongly on the size of the samples, where shorter samples cause a greater decrease in the temperature of the cooling (due to the diffusion of heat by transporters) compared to the longer samples. Transient heat transport analysis for pulse operation in semiconductors provides additional information about the optimum geometric parameters which can facilitate the design of thermoelements. In this manner, knowing the characteristic cooling length of a thermoelement could be the principle needed to make a judgment on whether optimum geometric parameters exist to achieve a lowest cooling temperature.

**Figure 5:**
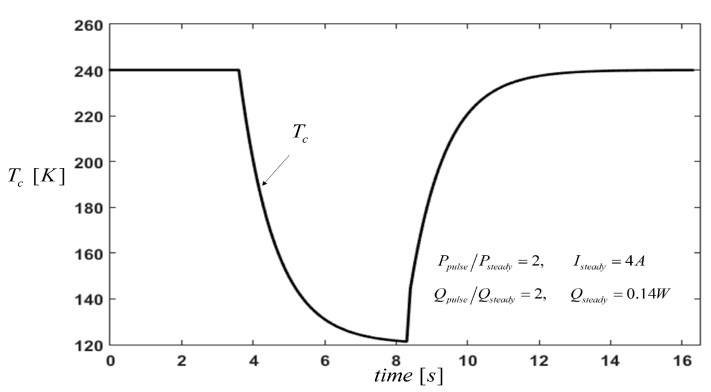
Cold side temperature of the thermoelement with pulse operation.

Figure 5 shows the transient behaviour of the temperature $T_{c}$ during a pulse current and pulse cooling load operation. The maximum temperature drop, $\Delta T_{max}=80$ K, was archived without considering the impacts of the metal strips and ceramic plates. This value of $\Delta T_{max}$ was because the Thomson effect was proportional to the current, while Joule heating is proportional to the square of current. As a result, once the value of the current is elevated, the increase in the temperatures’ differences of the P-type element are higher. Thus, when a TEM experiences a current pulse, the cooling at the cold junction occurs before the Joule heat reaches the cold end, and finally achieves a $T_{c}$ drop greater than that obtained by a steady operation mode.

### 4.2. Temperature Profile: Variable Pulse of Cooling Load

**Figure 6:**
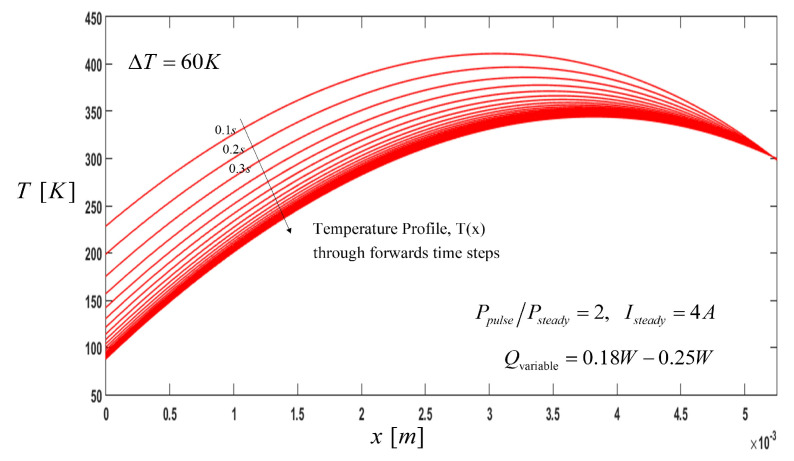
Temperature Profile, T(x): Effect of the pulse current and variable pulse cooling load along the thermoelement, where the direction of the arrow indicates the temperature profile through the time steps until reaching the steady state.

Under different cooling loads, the thermal behavior of the thermoelectric cooler could be different. The effects of variable cooling loads during pulse operation on thermal behavior is shown in Figure 6. Lower temperature values are obtained, $T_{C,min}=87.74$ K, compared to a constant pulse cooling load reducing, as well as increasing the characteristic cooling length.

**Figure 7:**
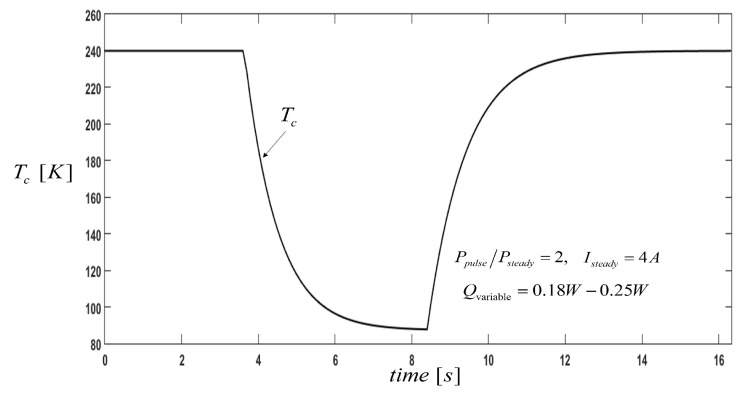
Cold side temperature of the thermoelement with variable pulse operation.

Figure 7 shows the transient temperature $T_{C}$ during pulse operation, and notably, there exists obvious supercooling in the various cases with variable $Q_{c}$ values. The cold side temperature of the thermoelectric cooler decreases with the decrease in pulse cooling load, because at higher currents the thermoelectric cooler can provide more cooling loads for a fixed cold side temperature. However, in this case, the operating current increases (pulse current) and the cooling load is low, which leads to the decrease in the cold side temperature.

It is well-known that transient supercooling is strongly dependent on the material and structure parameters, such as the leg length and the cross-sectional area of semiconductor. Numerical modeling and simulations are very important tools which are currently used to investigate the supercooling effect in TECs, but in previous studies, the Thomson heat has very often been ignored and only the Joule heating was assumed to be the internal thermal source. As established above, a multiphysics model using a 2D approach is indeed needed to accurately simulate the device performance and to show transient temperature distribution along the thermoelement.

## 5. Conclusions

This paper presented an investigation of a TEC semiconductor element considering a transient cooling operation, and the corresponding novel MATLAB code model was developed. Equations for the numerical method based on the finite element analysis were developed and applied in MATLAB programming. The simulation model is used to a parametric study which investigate the effects of the parameters such as semiconductor geometry, pulse electrical current, and cooling load on the minimum cooling temperature achievable. A ratio $P=I_{pulse}/I_{steady}=2$, with $I_{steady}=2$ A, and a cooling load ratio of $Q=Q_{C,pulse}/Q_{C,steady}=2.3$, with $Q_{c,steady}=0.15$ W, were used in all simulations considering thermoelectric constant properties of the material semiconductor.

From Equation (1) we can see that Joule and Thomson terms can be considered as a source and sink terms, respectively. Therefore, in general, Joule heating leads to an increase in the hot side temperature, whereas the Thomson effect tends to decrease the cold side temperature. The electric flow is higher for pulsed-like transport as a consequence of concentrating the charge carriers. The temperature difference between the hot and cold ends increases with the applied current, and we also found that the temperature difference between the two ends increased with *L*. As *L* is changed from 4.75 to 3.5$\times 10^{-3}$ m, the value of $T_{c}$ reduced from approximately 142 to 90 K. The temperature of the cold ends increased with cooling load $Q_{c}$; thus, high values of Q are desirable. Since the average cold side temperature Tc increases with the increase in $Q_{c}$, the optimum pulse operation can be selected based on the acceptable average cold side temperature. Cooling temperature decreases even more when a variable pulse cooling load is considered, in addition to an electrical current pulse that is normally used to reach minimum cold side temperature values compared to steady-state operation.

According to the results, we propose a general principle that the lowest cooling temperature values occur for minimum values of the semiconductor length and for a variable pulse cooling load compared to that under the steady-current operation. This last statement can be explained thanks to a characteristic cooling length, which is a characteristic in the material that can be observed only during pulse current operation and depends on geometric parameters, such as semiconductor length. This work is an important basis for future works on the development 2D models and investigations on the effect of geometries in the temperature overshoot of the cold side temperature. This work also provides a methodology to establish a new parameter, characteristic cooling length, and conditions which affect the cooling to provide an important perspective into the characteristics of the temperature overshoot, and contributes to the optimum design of a realistic TEM under current pulse operations.

## Figures and Tables

**Table 1:** Properties of the thermoelectric materials.

Property	Material ${\mathrm{Bi}}_{2}{\mathrm{Te}}_{3}$	Unit
α	$210\times 10^{-6}$	V K${}^{-1}$
κ	1.363	W m${}^{-1}$ K${}^{-1}$
ϵ	1.05	Ωm
ρ	10,922.08	Kg m${}^{3}$
$T_{a}$	240	K
*A*	$12.25\times 10^{-6}$	m${}^{2}$
*L*	$4.25\times 10^{-3}$	m
$C_{p}$	200	J kg${}^{-1}$ K${}^{-1}$

## Abbreviation

*A*	Area (m${}^{2}$)
COP	Coefficient of performance
CPM	Constant material property
*I*	Electrical current (A)
$j_{el}$	Electrical current density (A m${}^{-2}$)
$j_{q}$	Heat flux (W m${}^{-2}$)
*L*	Length (m)
$Q_{c}$	Cooling power (W)
$Q_{h}$	Heat flow released (W)
T	Temperature (K)
$T_{c}$	Cold side temperature (K)
$T_{h}$	Hot side temperature (K)
TEC	Thermoelectric cooler
**Greek letters**	
α	Seebeck coefficient (V K${}^{-1}$)
Δ	Difference
κ	Thermal conductivity (W m${}^{-1}$K${}^{-1}$)
ϵ	Electrical resistivity [(Ωm)${}^{-1}$]
τ	Thomson coefficient [V K${}^{-1}$]
**Subscripts**	
i	local subdomain
min	Minimum values
max	Maximum values
pulse	appropriate for pulse operation
steady	appropriate for steady operation
